# Real-World Safety of Immune Checkpoint Inhibitors in Small Cell Lung Cancer: A Systematic Review of Comparative Cohort Studies

**DOI:** 10.1007/s11912-026-01774-7

**Published:** 2026-03-26

**Authors:** Juhee Koo, Chin Hang Yiu, Hieu T. Le, Kevin Winardi, Edwin C.K. Tan, Christine Y. Lu

**Affiliations:** 1 The University of Sydney School of Pharmacy, Camperdown, Sydney, NSW Australia; 2 Kolling Institute, Faculty of Medicine and Health, The University of Sydney and the Northern Sydney Local Health District, St Leonards, Sydney, NSW Australia; 3 Laboratory of Ageing and Pharmacology, Kolling Institute, Faculty of Medicine and Health, The University of Sydney and the Northern Sydney Local Health District, St Leonards, Sydney, NSW Australia; 4 Department of Pharmacy, Royal North Shore Hospital, St Leonards, Sydney, NSW Australia

**Keywords:** Small cell lung cancer, Immune checkpoint inhibitors, Real-world data, Comparative safety, Systematic review

## Abstract

**Introduction:**

Immune checkpoint inhibitors (ICIs) have significantly improved survival outcomes in small cell lung cancer (SCLC). However, ICIs can cause treatment-related adverse events (TRAEs), particularly immune-related adverse events (irAEs), which may compromise treatment efficacy and patient quality of life. Most ICI safety data is derived from clinical trials, which may not reflect real-world populations. This systematic review evaluated the real-world safety of ICIs compared to other therapies in SCLC.

**Methods:**

A systematic search of MEDLINE, Embase, Scopus, CINAHL was conducted from inception to July 21, 2025. Eligible studies were observational cohort studies reporting safety outcomes for ICIs versus other cancer therapies (e.g., chemotherapy, targeted therapy) in SCLC. Study quality was assessed using the Newcastle-Ottawa Scale. A narrative synthesis was conducted to summarise key findings.

**Results:**

Twenty retrospective cohort studies were included, all using electronic health record data. Cohort sizes ranged from 14 to 188 patients. Nineteen studies were rated as poor quality due to inadequate adjustment for confounding variables. Across studies, TRAE incidence was comparable between ICI plus chemotherapy combination and chemotherapy alone. Similarly, TRAE and irAE rates were consistent across different ICI plus chemotherapy regimens. Evidence comparing ICIs to targeted therapy was limited.

**Conclusion:**

Real-world evidence suggests that adding ICIs to chemotherapy does not substantially increase toxicity in patients with SCLC, and safety profiles are generally consistent across ICI regimens. However, findings are constrained by small sample sizes and poor methodological quality. High-quality, large-scale observational studies are needed to validate these results and better inform clinical decision-making.

**Supplementary Information:**

The online version contains supplementary material available at 10.1007/s11912-026-01774-7.

## Introduction

Lung cancer remains the most frequently diagnosed cancer and the leading cause of cancer-related deaths worldwide, accounting for an estimated 2.5 million new cases and 1.8 million deaths in 2022 [1]. Small cell lung cancer (SCLC) represents approximately 10–15% of lung cancer cases and is the most aggressive subtype, with a 5-year survival rate of just 9%, compared to 32% in non-small cell lung cancer (NSCLC) [2]. Due to its rapid proliferation and early metastatic spread, the majority of SCLC patients are diagnosed with extensive-stage disease, resulting in limited treatment options and poor prognosis [3].

For over four decades, platinum-based chemotherapy has remained the standard first- and second-line treatment for SCLC. While it achieves high initial response rates, these are typically short-lived, with most patients relapsing quickly and developing resistance to further lines of therapy [4]. In recent years, immune checkpoint inhibitors (ICIs) have emerged as a major advancement in oncology. These therapies enhance antitumour immunity by inhibiting immune checkpoints such as cytotoxic T-lymphocyte antigen 4 (CTLA-4) and programmed cell death 1/ programmed cell death ligand 1 (PD-1/PD-L1) [5].

Nivolumab was the first ICI approved for SCLC by the U.S. Food and Drug Administration (FDA) in 2018, followed by atezolizumab’s approval by the European Medicines Agency (EMA) in 2019 [6, 7]. Landmark trials such as IMpower133 [8] and ADRIATIC [9] demonstrated that adding ICIs to standard chemotherapy improved overall survival in both extensive-stage (ES-SCLC) and limited-stage (LS-SCLC) disease, with survival gains of approximately 2 months in ES-SCLC and up to 22 months in LS-SCLC. As a result, ICIs, particularly atezolizumab and durvalumab, have been incorporated into first-line therapy for ES-SCLC, while durvalumab has also been incorporated as consolidation (maintenance) therapy for LS-SCLC [10, 11]. Although some early ICIs were discontinued due to limited efficacy, ongoing clinical trials continue to explore their therapeutic potential in SCLC [12, 13].

Despite these advances, ICIs are associated with treatment-related adverse events (TRAEs), particularly immune-related adverse events (irAEs), which can compromise treatment efficacy and patient quality of life [14]. IrAEs represent a distinct class of toxicities that mimic autoimmune conditions and may affect any organ system, with the skin (rash, pruritus), gastrointestinal tract (diarrhoea, colitis), lungs (pneumonitis), liver (hepatitis), and endocrine glands (hypothyroidism, adrenal insufficiency) being most commonly involved [15, 16]. Moderate to severe irAEs can lead to treatment discontinuation, long-term organ dysfunction, hospitalisation, and, in some cases, mortality, reported in up to 1.3% of patients [15, 17]. The cumulative burden of irAE management, including hospital care, immunosuppressive treatment, and monitoring, also contributes significantly to rising healthcare costs [18, 19]. Therefore, clinician awareness and proactive management of irAEs are critical to ensuring optimal clinical and economic outcomes.

To date, most safety data on ICIs have been derived from randomized controlled trials (RCTs), which, although methodologically rigorous, often include highly selected patient populations that may not reflect real-world clinical practice [20]. Existing systematic reviews examining the safety of ICIs in SCLC are largely based on RCTs and may not account for the broader heterogeneity seen in routine care [21–23]. Real-world data, sourced from electronic health records, administrative databases, or cancer registries, offer valuable insights by capturing more diverse patient populations, including those with comorbidities, poor performance status, or concomitant therapies [20, 24–26]. However, most real-world studies of ICI safety in lung cancer have focused predominantly on NSCLC, leaving SCLC relatively underexplored despite its more aggressive clinical trajectory and unique treatment challenges [27, 28].

Given these limitations, there is a growing need for real-world evidence to better characterise the safety profile of ICIs in broader, more clinically representative SCLC populations. Therefore, this systematic review evaluates the real-world safety of ICI-based regimens compared to other systemic therapies, including chemotherapy, targeted therapy, and alternative ICI combinations, in patients with SCLC. By focusing specifically on this underrepresented subgroup, the review aims to address a key gap in the literature and support more-informed clinical decision-making.

## Method

This systematic review was reported in accordance with the Preferred Reporting Items for Systematic Reviews and Meta-analyses (PRISMA) guidelines [29]. The protocol was prospectively registered on PROSPERO (CRD420251046722) and any protocol changes, along with their justifications, are detailed in Supplementary Table S1.

### Search Strategy

A comprehensive literature search was conducted across four databases: MEDLINE, Embase, Scopus, and CINAHL, from database inception to July 21, 2025, with no language or date restrictions. Search terms combined key concepts related to lung cancer, immune checkpoint inhibitors, adverse events, and real-world data (e.g., electronic health records, medical claims data, and cancer registries). The full search strategy is available in Supplementary Table S2.

### Inclusion and Exclusion Criteria

Eligible studies were real-world observational cohort studies evaluating the use of ICIs in patients with SCLC, with no restrictions on cancer stage, treatment line, prior therapy, or follow-up duration. Any ICI regimen (e.g., monotherapy, ICI plus chemotherapy, ICI plus targeted therapy) was eligible, provided it was compared against a pharmacological comparator group (e.g., chemotherapy, targeted therapy, or another ICI regimen) as our goal was to evaluate the comparative safety of ICIs. Studies were required to report at least one safety outcome, with no restrictions on type or severity.

The following were excluded: RCTs, case-control studies, case reports, literature reviews, study protocols, conference abstracts, and non-English publications. Case-control studies were excluded because they do not allow estimation of incidence rates, making them less suitable for safety assessments and limiting comparability across studies. Studies that did not clearly define the treatment administered, or reported aggregated outcomes across multiple therapies without stratification, were also excluded, as specific safety outcomes could not be attributed to individual therapies.

### Outcomes

This review assessed safety outcomes including the incidence of TRAEs, irAEs, unspecified adverse events (AEs), and treatment discontinuation, hospitalisations, or deaths due to toxicity. As comparator groups included non-ICI therapies, safety outcomes reported were not limited to irAEs.

### Study Selection

All search results were imported into Covidence (Veritas Health Innovation, Melbourne, Australia), and duplicates were removed [30]. Three reviewers (J.K., C.H.Y., and H.T.L.) independently screened titles and abstracts for eligibility. Full-texts of potentially eligible studies were independently assessed for final inclusion by the same reviewers. Disagreements were resolved through discussion and when necessary, consultation with a senior investigator (C.Y.L.), to reach consensus on eligibility.

### Data Extraction

Data extraction was performed independently by two reviewers (J.K. and C.H.Y.) using a pre-defined data extraction template. Any discrepancies were resolved through discussion and when necessary, consultation with a senior investigator (C.Y.L.), to reach a final decision. Extracted data included: study author, publication year, country, patient population, treatment groups, follow-up time, and reported safety outcomes.

### Data Synthesis

Due to heterogeneity in study designs, populations, follow-up durations, outcome definitions, and analytical approaches, a meta-analysis was not feasible. Therefore, a narrative synthesis was conducted to summarise key findings.

Adverse event reporting varied across studies; some reported only overall rates, while others provided incidence rates for specific event types. Where necessary, minor calculations were conducted to aggregate multiple reported events into an overall rate. For example, the incidence was calculated by dividing the total number of events by the sample size, yielding a per capita rate. An example calculation is presented in Supplementary Table S3.

To aid interpretation, studies were grouped broadly based on the type of safety outcome(s) reported; ‘Treatment-Related Adverse Events’ (TRAEs), ‘Immune-Related Adverse Events’ (irAEs), and ‘Unspecified Adverse Events’ (AEs). Adverse events explicitly described as treatment-related or drug-related were classified as TRAEs, and events were further classified as irAEs only if specifically identified as immune-related in the original study. Studies that did not specify whether adverse events were attributable to treatment were classified as unspecified AEs. As classification was based strictly on how adverse events were attributed in the original studies, the same toxicity (e.g., hematologic events) may appear in different categories across studies depending on whether treatment attribution was specified.

### Quality Assessment

The methodological quality of included studies was assessed using the Newcastle-Ottawa Scale (NOS), as all were non-randomised and non-interventional cohort studies. The NOS has been utilised in a systematic review of the real-world comparative efficacy and safety of ICIs in colorectal cancer [31]. Two authors (J.K. and C.H.Y.) independently evaluated each study across three domains: selection, comparability, and outcome assessment. Quality ratings were assigned as good, fair, or poor, based on the Agency for Healthcare Research and Quality (AHRQ) standards. Discrepancies were resolved through discussion or adjudication by a senior investigator (C.Y.L.).

## Results

**Fig. 1:**
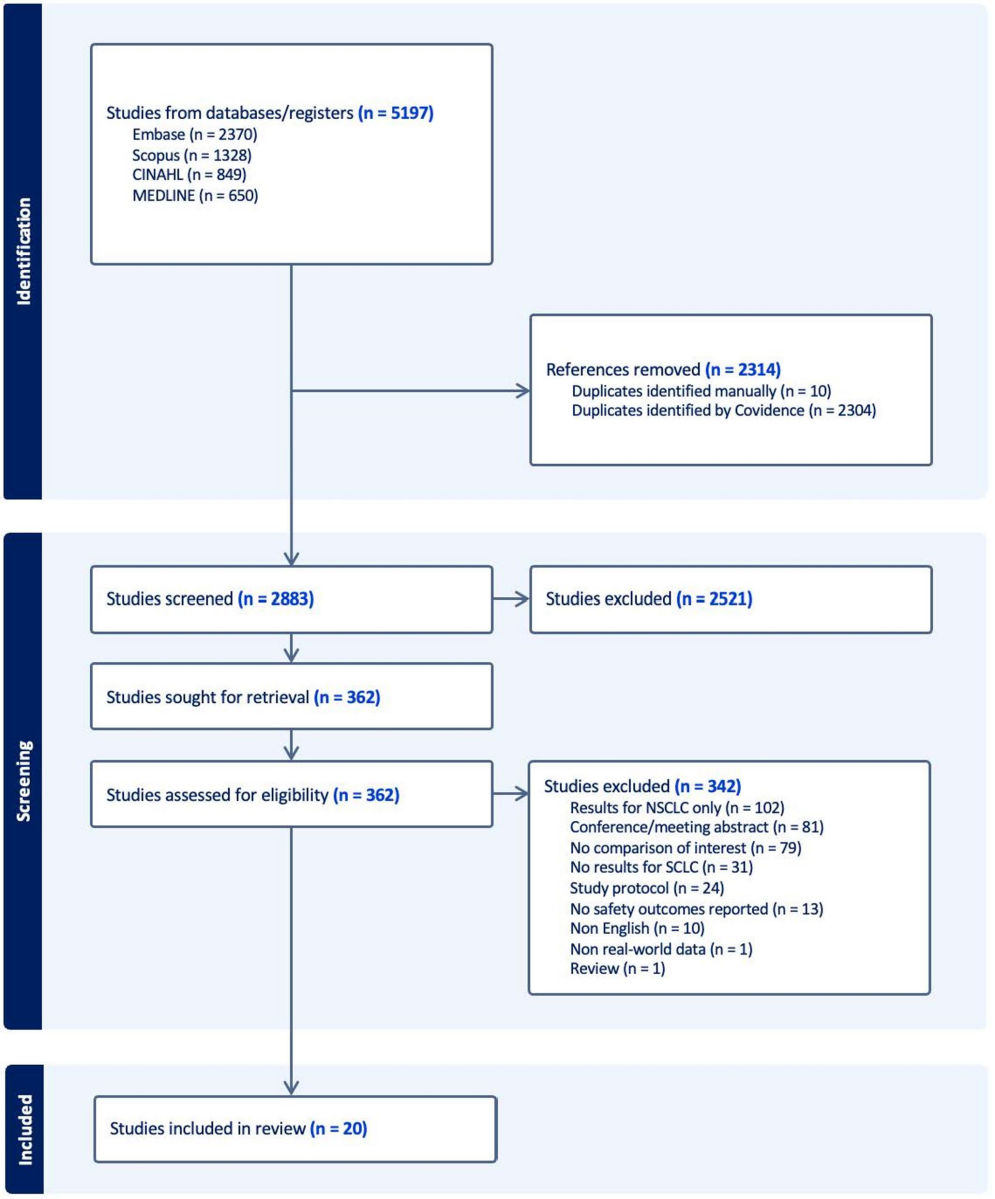
PRISMA flow diagram [29]. Abbreviations: NSCLC, non-small cell lung cancer; SCLC, small cell lung cancer

The database search generated 5,197 results. After removing duplicates, 2,883 records remained for screening. Following title and abstract screening, 2,521 studies were excluded. An additional 342 studies were excluded after full-text review, resulting in the inclusion of 20 studies in this review (Fig. 1) [32–51].

### Quality Assessment

Study quality assessments are presented in Supplementary Table S4. Only one study [38] was rated as good quality, as it employed propensity score matching, allowing for robust cohort comparability through balanced baseline characteristics [52]. The remaining 19 studies were rated as poor quality, primarily due to the lack of confounder adjustments [32–40, 42–51].

### Study Characteristics

Table 1 presents the key characteristics of the included studies. Most studies were conducted in China (*n* = 17) [32–45, 48, 50, 51], with one study each from the United States [47], France [49], and Japan [46]. All were retrospective cohort studies using safety data derived from patient electronic health records. In terms of disease stage, 15 studies included patients with ES-SCLC [33–36, 38–41, 43, 44, 46, 47, 49–51], two studies focused on LS-SCLC [37, 42], and three studies included patients with both ES- and LS-SCLC [32, 45, 48]. Collectively, the studies included 3,278 patients, with sample sizes ranging from 14 to 188 patients per cohort (median 59). Minimum reported follow-up durations ranged from 0.7 to 21 months. The ICIs investigated in the studies included adebrelimab, atezolizumab, camrelizumab, durvalumab, nivolumab, pembrolizumab, serplulimab, sintilimab, tislelizumab, and toripalimab. Most of these agents have either been approved for SCLC by the National Medical Products Administration (NMPA) in China, where the majority of studies were undertaken, or were used off-label in clinical settings [53–56]. To facilitate comparison, the 20 studies were categorised by the type of safety outcome reported: ‘Treatment-related adverse events’, ‘Immune-related adverse events’, and ‘Unspecified adverse events’ (Table 2). Four studies reported multiple types of safety outcomes and were therefore included in more than one category [34, 35, 44, 46].

**Table 1:** Characteristics of Included Studies (*n* = 20)

Author (year), Country	Study Group 1, Sample Size		Study Group 2, Sample Size	Safety Outcomes Investigated	Minimum Follow-Up Period
He et al. (2024), China [32]	ICI + Chemotherapy (*n* = 14)		Chemotherapy alone (*n* = 26)	TRAEs	21 months
Lamy et al. (2024), France [49]	ICI + Chemotherapy (*n* = 49)		Chemotherapy alone (*n* = 69)	AEs	12 months
Peng et al. (2025), China [33]	ICI + Chemotherapy (*n* = 86)		Chemotherapy alone (*n* = 64)	TRAEs	8 months
Qin et al. (2024), China [43]	Anti-PD-1 + Chemotherapy (*n* = 68)		Anti-PD-L1 + Chemotherapy (*n* = 86)	TRAEs	3 months
Qiu et al. (2023), China [34]	ICI + Chemotherapy (*n* = 165)		Chemotherapy alone (*n* = 188)	TRAEs	10 months
	Anti-PD-1 + Chemotherapy (*n* = 91)	Anti-PD-L1 + Chemotherapy (*n* = 74)		irAEs	
Qu et al. (2022), China [35]	ICI + Chemotherapy (*n* = 109)		Chemotherapy alone (*n* = 115)	TRAEs	2 months
	Atezolizumab + Chemotherapy (*n* = 68)	Durvalumab + Chemotherapy (*n* = 41)		irAEs	
Vince et al. (2024), America [47]	Atezolizumab + Chemotherapy (*n* = 46)		Durvalumab + Chemotherapy (*n* = 55)	irAEs	–
Wang et al. (2024), China [36]	Sintilimab + Chemotherapy (*n* = 24)		Chemotherapy alone (*n* = 22)	TRAEs	7 months
Wang et al. (2023), China [44]	Anti-PD-1 + Chemotherapy (*n* = 93)		Anti-PD-L1 + Chemotherapy (*n* = 101)	TRAEs and irAEs	8 months
Wan et al. (2023), China [50]	ICI + Chemotherapy (*n* = 27)		Chemotherapy alone (*n* = 29)	AEs	–
Xie et al. (2024), China [37]	ICI + Chemotherapy (*n* = 63)		Chemotherapy alone (*n* = 87)	TRAEs	21 months
	Anti-PD-1 + Chemotherapy (*n* = 45)	Anti-PD-L1 + Chemotherapy (*n* = 18)			
Xu et al. (2024), China [51]	ICI + Anlotinib (*n* = 19)		Anlotinib alone (*n* = 29)	AEs	2 months
Yamanaka et al. (2024), Japan [46]	Atezolizumab + Chemotherapy (*n* = 70)		Durvalumab + Chemotherapy (*n* = 30)	TRAEs and irAEs	0.7 months
J Zhang et al. (2025), China [38]	Cohort 1: ICI + Anlotinib (*n* = 86) Cohort 2: ICI + Chemotherapy (*n* = 125) Cohort 3: Chemotherapy alone (*n* = 178)			TRAEs	19 months
Q Zhang et al. (2025), China [45]	Anti-PD-1 + Chemotherapy (*n* = 19)		Anti-PD-L1 + Chemotherapy (*n* = 18)	TRAEs	–
Zhang et al. (2024), China [39]	Cohort 1: Anlotinib + Chemotherapy (*n* = 19) Cohort 2: Tislelizumab + Chemotherapy (*n* = 33) Cohort 3: Chemotherapy alone (*n* = 49)			TRAEs	–
Zhao et al. (2025), China [40]	ICI + Chemotherapy (*n* = 82)		Chemotherapy alone (*n* = 53)	TRAEs	18 months
Zhou et al. (2025), China [41]	ICI + Chemotherapy (*n* = 173)		Chemotherapy alone (*n* = 176)	TRAEs	9 months
Zhu et al. (2025), China [42]	ICI + Chemotherapy (*n* = 29)		Chemotherapy alone (*n* = 24)	TRAEs	12 months
Zou et al. (2023), China [48]	Atezolizumab + Chemotherapy (*n* = 43)		Durvalumab + Chemotherapy (*n* = 100)	irAEs	–

*Abbreviations*: *AE* Unspecified adverse event, *ICI* Immune checkpoint inhibitor, *irAE* Immune-related adverse event, *PD-1* Programmed cell death protein 1, *PD-L1* Programmed cell death ligand 1, *TRAE* Treatment-related adverse event

**Table 2:** Extracted safety outcomes of included studies (*n* = 20)

Author (year)	Incidence Reported
Treatment-Related Adverse Events (*n* = 15)	
He et al. (2024) [32]	Incidence rate of grade ≥ 3 TRAEs (*p* = 0.081) • ICI + Chemotherapy = 71.4% • Chemotherapy alone = 46.2%
Peng et al. (2025) [33]	Incidence rate of grade ≥ 3 TRAEs • ICI + Chemotherapy = 18.6% • Chemotherapy alone = 21.9% *No TRAEs leading to drug discontinuation or death in either group.*
Qiu et al. (2023) [34]	Incidence rate of grade 3–4 TRAEs (*p* = 0.186) • ICI + Chemotherapy = 10.91% • Chemotherapy alone = 6.91%
Qu et al. (2022) [35]	Incidence rate of grade 3–4 TRAEs • ICI + Chemotherapy = 45.9% • Chemotherapy alone = 41.7% Death related to TRAEs • ICI + Chemotherapy = 2.8% • Chemotherapy alone = 4.3%
Wang et al. (2024) [36]	Incidence rate of grade 3–5 TRAEs (*p* = 0.69) • Sintilimab + Chemotherapy = 12.5% • Chemotherapy alone = 18.2%
Xie et al. (2024) [37]	Incidence rate of grade 3–4 TRAEs (*p* = 0.62) • ICI + Chemotherapy = 49.1% o PD-1 + Chemotherapy = 48.7% (*p* = 0.56) o PD-L1 + Chemotherapy = 50.0% • Chemotherapy alone = 42.5%
J, Zhang et al. (2025) [38]	Incidence rate per capita of grade 3–4 TRAEs • ICI + Chemotherapy = 0.12 • Chemotherapy alone = 0.19 • ICI + Anlotinib = 0.13 *No treatment-related deaths occurred in either group.*
Zhang et al. (2024) [39]	Incidence rate of grade ≥ 3 TRAEs • Tislelizumab + Chemotherapy = 36.4% • Chemotherapy alone = 32.7% • Anlotinib + Chemotherapy = 31.6%
Zhao et al. (2025) [40]	Incidence rate per capita of grade ≥ 3 TRAEs • ICI + Chemotherapy = 0.96 • Chemotherapy alone = 0.77
Zhou et al. (2025) [41]	Incidence rate of grade 3–4 TRAEs • ICI + Chemotherapy = 24.3% • Chemotherapy alone = 18.2%
Zhu et al. (2025) [42]	Incidence rate of grade ≥ 3 TRAEs (*p* > 0.05) • ICI + Chemotherapy = 6.9% • Chemotherapy alone = 29.2%
Qin et al. (2024) [43]	Incidence rate per capita of grade ≥ 3 TRAEs • Anti-PD-1 + Chemotherapy = 0.59 • Anti-PD-L1 + Chemotherapy = 0.62 Treatment discontinuation due to TRAEs • Anti-PD-1 + Chemotherapy = 4.4% • Anti-PD-L1 + Chemotherapy = 8.1% *No treatment-related deaths occurred in either group.*
Wang et al. (2023) [44]	Incidence rate of grade 3–4 TRAEs • Anti-PD-1 + Chemotherapy = 33.3% • Anti-PD-L1 + Chemotherapy = 33.7% Treatment discontinuation due to TRAEs • Anti-PD-1 + Chemotherapy = 4.3% • Anti-PD-L1 + Chemotherapy = 2.0% Death related to TRAEs • Anti-PD-1 + Chemotherapy = 2.2% • Anti-PD-L1 + Chemotherapy = 1.0%
Q, Zhang et al. (2025) [45]	Incidence rate per capita of any-grade TRAEs • Anti-PD-1 + Chemotherapy = 2.63 • Anti-PD-L1 + Chemotherapy = 2.0
Yamanaka et al. (2024) [46]	Incidence rate per capita of grade ≥ 3 TRAEs • Atezolizumab + Chemotherapy = 1.1 • Durvalumab + Chemotherapy = 1.5
Immune-Related Adverse Events (*n* = 6)	
Qiu et al. (2023) [34]	Incidence rate of grade 3–4 irAEs (*p* = 0.785) • Anti-PD-1 + Chemotherapy = 52.38% • Anti-PD-L1 + Chemotherapy = 47.62%
Wang et al. (2023) [44]	Incidence rate of grade 3–4 irAEs • Anti-PD-1 + Chemotherapy = 5.4% • Anti-PD-L1 + Chemotherapy = 5.0%
Qu et al. (2022) [35]	Incidence rate of grade 3–4 irAEs • Atezolizumab + Chemotherapy = 7.4% • Durvalumab + Chemotherapy = 4.9%
Vince et al. (2024) [47]	Incidence rate of any-grade irAEs (*p* = 0.157) • Atezolizumab + Chemotherapy = 47.8% • Durvalumab + Chemotherapy = 32.7% Incidence rate of hospitalisations due to irAEs (*p* = 0.204) • Atezolizumab + Chemotherapy = 36.4% • Durvalumab + Chemotherapy = 17.6% Treatment discontinuation due to irAEs (*p* = 0.221) • Atezolizumab + Chemotherapy = 9.1% • Durvalumab + Chemotherapy = 22.2%
Yamanaka et al. (2024) [46]	Incidence rate of grade 3–4 irAEs • Atezolizumab + Chemotherapy = 17% • Durvalumab + Chemotherapy = 20%
Zou et al. (2023) [48]	Incidence rate of grade 3–4 irAEs • Atezolizumab + Chemotherapy = 2.3% • Durvalumab + Chemotherapy = 10.0% Treatment discontinuation due to irAEs (*p* = 0.79) • Atezolizumab + Chemotherapy = 4.7% • Durvalumab + Chemotherapy = 10.0% *No treatment-related deaths occurred in either group.*
Unspecified Adverse Events (*n* = 3)	
Lamy et al. (2024) [49]	Incidence rate of grade 3–4 AEs (*p* = 0.003) • ICI + Chemotherapy = 18.4% • Chemotherapy alone = 44.9% Treatment discontinuation due to AEs • ICI + Chemotherapy = 9.7% • Chemotherapy alone = 23.2% Death related to AEs • ICI + Chemotherapy = 3.2% • Chemotherapy alone = 8.7%
Wan et al. (2023) [50]	Incidence rate per capita of grade ≥ 3 AEs • ICI + Chemotherapy = 0.11 • Chemotherapy alone = 0.10
Xu et al. (2024) [51]	Incidence rate per capita of any-grade AEs • ICI + Anlotinib = 1.16 • Anlotinib alone = 0.86

*Abbreviations*: *AE* Unspecified adverse event, *ICI* Immune checkpoint inhibitor, *irAE* Immune-related adverse event, *PD-1* Programmed cell death protein 1, *PD-L1* Programmed cell death-ligand 1, *TRAE* Treatment-related adverse event

#### Treatment-Related Adverse Events (TRAEs)

**Fig. 2:**
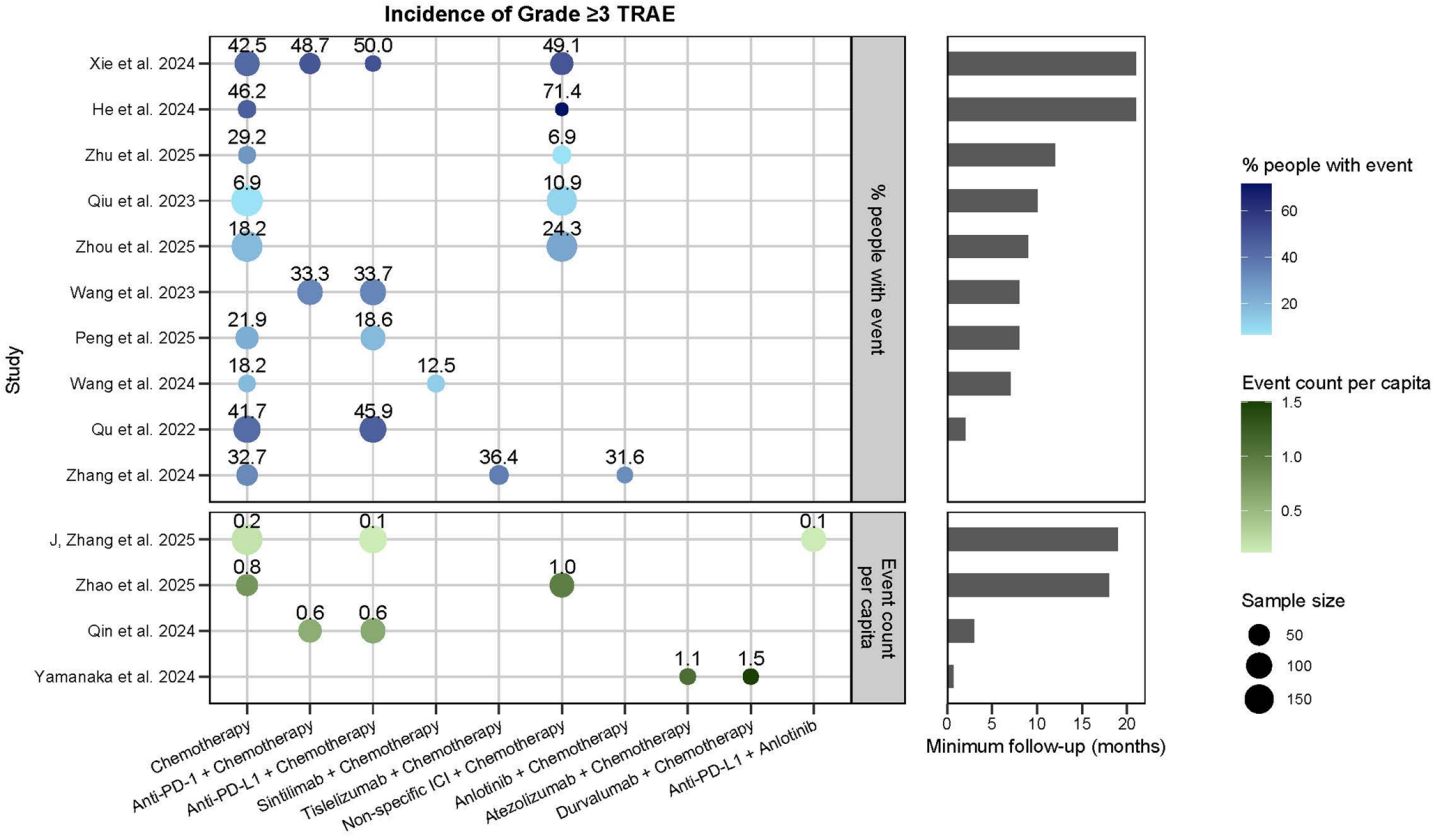
Incidence of grade ≥ 3 TRAEs and minimum follow-up times (months) across 14 studies. Each bubble represents a study reporting grade ≥ 3 TRAEs, stratified by treatment regimen. The x-axis indicates treatment regimen, and the y-axis lists individual studies. Bubble size reflects study sample size, and numeric labels above bubbles show the reported incidence (%) or event count per capita. The top panel shows the percentage of patients experiencing grade ≥ 3 TRAEs (colour scale: light to dark blue = lower to higher incidence). The bottom panel depicts event count per capita (colour scale: light to dark green = lower to higher count). Horizontal bar plots on the right display the minimum follow-up duration (months) for each study. Abbreviations: ICI, immune checkpoint inhibitor; PD-1, programmed cell death protein 1; PD-L1, programmed cell death ligand 1; TRAE, treatment-related adverse event

A total of 15 studies reported on TRAEs [32–46], of which 14 provided data on grade ≥ 3 TRAEs (Fig. 2); Zhang et al., reported only any-grade TRAEs [45]. 11 studies compared ICI plus chemotherapy versus chemotherapy alone, and all reported no statistically significant difference in the overall incidence of TRAEs between the two groups [32–42]. Notably, the only good-quality study by Zhou et al. (*n* = 349) [41], which utilised propensity score matching to control for baseline confounders such as age and medical history, also found no significant difference in TRAE incidence between ICI plus chemotherapy and chemotherapy alone (83.8% vs. 79%, *p* = 0.246). Across these studies, the most commonly reported TRAEs were haematological toxicities (e.g., neutropenia, leukopenia, thrombocytopenia, anaemia) as well as nausea and vomiting, with similar frequencies in both groups.

Five studies compared TRAEs across different ICI regimens combined with chemotherapy [37, 43–46]. Four of these evaluated PD-1 inhibitors versus PD-L1 inhibitors, and all reported comparable overall TRAE incidence between the two groups [37, 43–45]. In a study by Yamanaka et al. [46], two PD-L1 inhibitors, atezolizumab and durvalumab, were compared in combination with chemotherapy. The incidence of TRAEs per capita did not significantly differ between the two regimens (1.1 vs. 1.5, respectively).

Only two studies compared TRAEs involving targeted therapy-based regimens as a comparator [38, 39]. One study found similar TRAE incidence between patients receiving ICI plus chemotherapy and those receiving ICI plus targeted therapy, with no TRAE-related death reported in either group [38]. The other study compared ICI plus chemotherapy, targeted therapy plus chemotherapy, and chemotherapy alone, and likewise found no significant differences in TRAE incidence across these combinations [39].

#### Immune-Related Adverse Events (irAEs)

**Fig. 3:**
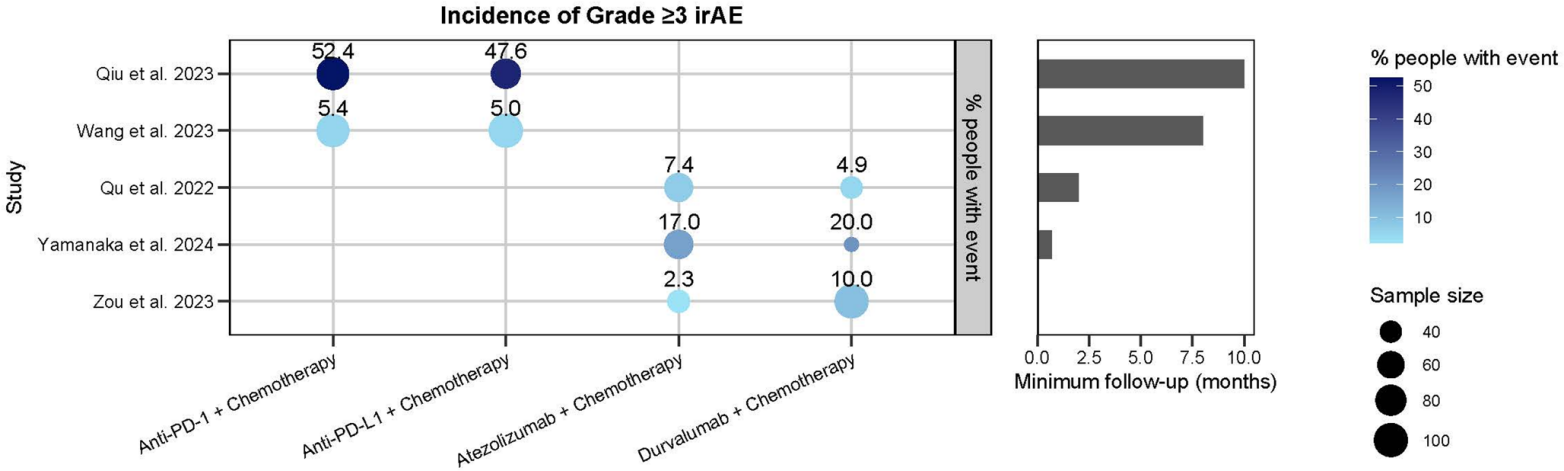
Incidence of grade ≥ 3 irAEs and minimum follow-up times (months) across 5 included studies. Each bubble represents a study reporting grade ≥ 3 irAEs, stratified by treatment regimen. The x-axis indicates treatment regimen, while the y-axis lists individual studies. Bubble size reflects study sample size, and numeric labels above bubbles denote the reported incidence rate. Bubbles are colour-coded by incidence (light blue = lower incidence; dark blue = higher incidence). Horizontal bar plots on the right display the minimum follow-up duration (months) for each study. Abbreviations: ICI, immune checkpoint inhibitor; PD-1, programmed cell death protein 1; PD-L1, programmed cell death ligand 1; irAE, immune-related adverse event

Six studies reported on irAEs [34, 35, 44, 46–48], all of which compared irAE incidence between different ICIs administered in combination with chemotherapy. Five of these studies reported grade ≥ 3 irAEs (Fig. 3), while Vince et al., reported only any-grade irAEs [47]. Four studies directly compared atezolizumab and durvalumab, both in combination with chemotherapy, and consistently found no significant difference in irAE incidence between the two regimens [35, 46–48]. Among these, two studies also assessed treatment discontinuation and hospitalisation due to irAEs and reported no statistically significant differences in these outcomes between the two groups [47, 48]. Across all studies, the most frequently reported irAEs were pulmonary toxicity (e.g., pneumonitis), cutaneous toxicity (e.g., rash, pruritus), and endocrine dysfunction, particularly thyroid dysfunction.

Two additional studies compared PD-1 inhibitors with PD-L1 inhibitors in combination regimens and similarly found no significant differences in irAE incidence [34, 44]. In one of the larger studies, Qiu et al., (*n* = 353) reported irAE rates of 52.38% for the PD-1 group versus 47.62% in the PD-L1 group (*p* = 0.785) [34].

#### Unspecified Adverse Events (AEs)

Three studies reported on AEs without specifying whether they were treatment-related or immune-related [49–51]. Of these, two studies compared ICI plus chemotherapy versus chemotherapy alone [49, 50]. One study reported no significant difference in the incidence of any-grade or grade 3–4 AEs between the two groups [50]. In contrast, a study by Lamy et al. [49], found a significantly higher incidence of AEs in the chemotherapy alone group (44.9%) compared to the ICI plus chemotherapy group (18.4%) (*p* = 0.003), as well as higher rates of treatment discontinuation due to AEs (23.2% vs. 9.7%). Across both studies, the most frequently reported AEs in both ICI plus chemotherapy and chemotherapy alone groups were haematological AEs (e.g., neutropenia, myelosuppression, anaemia).

The third study compared ICI plus targeted therapy with targeted therapy alone [51]. The incidence of AEs per capita was 1.16 in the ICI plus targeted therapy group and 0.86 in the targeted therapy alone group. The most common AEs reported in both groups were hypertension and hand-foot skin reaction.

## Discussion

To the best of our knowledge, this is the first systematic review to synthesise real-world evidence from cohort studies comparing the safety of ICIs with other therapies in patients with SCLC. Findings from this review suggest that combining ICIs with chemotherapy does not increase the incidence of TRAEs beyond those observed with chemotherapy alone. This observation aligns with results from the pivotal IMPower133 trial [8], which reported a comparable incidence of grade 3–4 TRAEs between the ICI plus chemotherapy group (56.6%) and the chemotherapy alone group (56.1%). In contrast to TRAEs, the evidence regarding all-cause AEs between ICI plus chemotherapy and chemotherapy alone remains inconclusive, owing to conflicting results and the limited number of relevant real-world studies (*n* = 2). One study [50] was consistent with findings from the KEYNOTE-604 trial [57], which found similar rates of grade 3–4 all-cause AEs in the ICI plus chemotherapy and chemotherapy alone arms (76.7% vs. 74.9%, respectively). However, the second real-world study [49] reported a higher AE incidence in the chemotherapy alone group than in the ICI plus chemotherapy group. This difference may reflect variation in event attribution, small sample sizes (*n* = 56 and *n* = 118), and the absence of confounder adjustments [49, 50], rather than a true reduction in toxicity with combination therapy. Across both real-world studies and landmark trials, haematologic toxicities were the most commonly reported AEs and TRAEs in both treatment groups, consistent with the known safety profile of platinum-based chemotherapy.

Our results also indicate that ICI plus chemotherapy combinations, irrespective of the specific ICI used, generally demonstrate comparable safety profiles, with similar incidences of both TRAEs and irAEs. These findings are consistent with a recent systematic review of landmark RCTs by Chen et al., which also reported similar TRAE rates across different ICIs [58]. However, their analysis identified a higher risk of irAEs among patients treated with durvalumab compared to those receiving atezolizumab (odds ratio [OR] 0.22, 95% CI 0.10–0.50), and compared to pembrolizumab (OR 3.12, 95% CI 1.27–7.64) [58]. This difference was not observed in our review, likely due to limited sample sizes, generally poor study quality and underreporting and inconsistent assessment of irAEs in real-world settings [16]. Nonetheless, the incidence of irAEs observed across ICI plus chemotherapy combinations in our review remains clinically relevant, particularly given emerging evidence suggesting a possible association between irAE occurrence and treatment efficacy. Several studies, including those specific to SCLC, have reported improved survival outcomes in patients who develop irAEs while receiving ICIs [59, 60]. As irAEs are thought to reflect immune activation, the similar incidence observed between PD-1 and PD-L1 inhibitors in our review may suggest broadly comparable immunologic effects, although direct comparative data are needed to determine whether this translates into equivalent clinical efficacy. Despite similar toxicity profiles, all PD-1 inhibitors evaluated for SCLC (e.g., nivolumab and pembrolizumab) have had their FDA SCLC indications withdrawn due to insufficient efficacy, whereas PD-L1 inhibitors (atezolizumab and durvalumab) remain approved for this indication [12, 13]. Therefore, our findings do not support a definitive association between irAE incidence and ICI efficacy in SCLC, highlighting the need for further investigation into this relationship.

Comparative safety data involving targeted therapy were less conclusive due to the limited number of studies identified, with only two reporting on TRAEs and one on AEs, each involving different regimens. Furthermore, the only targeted therapy assessed was anlotinib. The paucity of data likely reflects the limited and still emerging role of targeted therapy in SCLC treatment, with anlotinib only recently approved by the NMPA in China [61]. Although not adopted into routine practice outside of China, preliminary data presented at ASCO annual meetings have shown encouraging outcomes [62], and several ongoing small-scale RCTs are exploring targeted therapy regimens, particularly in combination with ICIs for SCLC [63, 64]. While these approaches remain investigational, understanding their comparative safety in real-world settings may be informative. Nevertheless, larger and more robust, head-to-head real-world studies are needed to determine which regimens (ICIs, targeted therapies, or their combinations) offer the most favourable safety profiles in clinical practice.

### Strengths and Limitations

A major strength of this systematic review is the comprehensive and methodologically rigorous search strategy, which included four major electronic databases to capture relevant real-world evidence. In addition, this review uniquely examines adverse event incidence across a wide range of ICI-based regimens, offering a broader and more nuanced understanding of the safety profiles of ICIs in SCLC. Importantly, it focuses on SCLC patients, an underrepresented group in the literature, as most existing studies concentrate on NSCLC populations [65].

However, the review is subject to several limitations. First, the heterogeneity in study designs, populations, comparator groups, and outcome definitions precluded meta-analysis. Second, the overall quality of the included studies was low, with 19 out of 20 rated as poor due to small sample sizes and inadequate adjustment for confounding variables. Third, the inclusion of both LS-SCLC and ES-SCLC populations introduces clinical heterogeneity, as differing treatment regimens between stages may influence adverse event incidence and limit definitive conclusions [10, 66]. Fourth, the geographic concentration of studies in China limits generalisability, particularly as some of the ICIs studied are not approved outside that region. Furthermore, variations in how TRAEs and irAEs were defined and reported across studies, often lacking standardised grading or attribution, introduce uncertainty and limit the interpretability of the aggregated findings. As a result, cross-study comparisons must be interpreted with caution.

This systematic review also highlights several methodological gaps in current real-world research. Most studies evaluated ICIs as part of combination regimens, limiting our ability to assess the safety of ICIs used as monotherapy. While this reflects real-world clinical practice in advanced SCLC [10], it underscores the need for real-world comparative studies that also examine monotherapy use. Additionally, many studies failed to perform appropriate confounder adjustments, weakening the robustness of their conclusions. Future research should incorporate these adjustments, such as through propensity score matching, to improve validity. Finally, standardised definitions, transparent reporting of adverse event grading systems, and multi-reviewer adjudication processes should be prioritised to ensure higher consistency and reliability in safety outcome reporting [67].

## Conclusion

To our knowledge, this is the first systematic review to evaluate the real-world safety of ICIs compared with other therapies in patients with SCLC. The results suggest that combining ICIs with chemotherapy does not appear to increase the incidence of TRAEs beyond that seen with chemotherapy alone, aligning with findings from landmark clinical trials. Additionally, across different ICI agents used in combination regimens, the incidence of both TRAEs and irAEs appear broadly comparable. However, the comparative safety of targeted therapies remain less well-defined due to a limited number of available studies. Moreover, the lack of head-to-head comparisons involving ICI monotherapy precludes firm conclusions regarding the relative safety of different therapeutic approaches. Given the small sample sizes, geographic concentration, and overall poor quality of included studies, there is a pressing need for larger, high-quality, real-world research to better define the safety of ICI-based regimens in SCLC. Strengthening this evidence base will be critical to inform treatment decisions and optimise patient outcomes in clinical practice.

## Key References

### Of Outstanding Importance

- Zhou J-X, Sun Y-C, Xiao L, Lu H-L, Yin X-M, Fan K, et al. Efficacy analysis and prognostic factors of first-line chemotherapy combined with immunotherapy in extensive-stage small cell lung cancer: a real-world study. Sci Rep. Nature Publishing Group; 2025;15:13063. 10.1038/s41598-025-98018-8
  - ○ This is the only included real-world study in this review that was rated as good-quality (*n* = 349). There was no significant difference in TRAE incidence between ICI + Chemotherapy and Chemotherapy alone.

### Of Importance

- He Y, Kong L, Ji X, Zhuo M, An T, Jia B, et al. Women patients with small-cell lung cancer using immunotherapy in a real-world cohort achieved long-term survival. Thorac Cancer. 2024;15:1727–38. 10.1111/1759-7714.15393.
  - ○ This study on 87 patients has a long minimum follow-up time of 21 months and reports no significant difference in the rate of grade ≥ 3 TRAEs between ICI plus Chemotherapy and Chemotherapy alone.
- Qiu G, Wang F, Xie X, Liu T, Zeng C, Chen Z, et al. A retrospective real-world experience of immunotherapy in patients with extensive stage small-cell lung cancer. Cancer Med. 2023;12:14881–91. 10.1002/cam4.5843.
  - ○ This real-world study (*n* = 353) found no significant difference in the incidence of grade ≥ 3 TRAEs between ICI plus Chemotherapy and Chemotherapy alone. It also showed similar rates of grade ≥ 3 irAEs between Anti-PD-1 and Anti-PD-L1 inhibitors when combined with chemotherapy.
- Wang Y, Li L, Hu J, Zhao Y, Yan H, Gao M, et al. Comparison of efficacy and safety between PD-1 inhibitors and PD-L1 inhibitors plus platinum-etoposide as first-line treatment for extensive-stage small-cell lung cancer: a multicenter, real-world analysis. BMC Cancer. 2023;23:1196. https://doi.org/10.1186/s12885-023-11709-1.
  - ○ This real-world study with 194 participants observed no significant variation in grade ≥ 3 TRAEs and irAEs between Anti-PD-1 and Anti-PD-L1 inhibitors when combined with chemotherapy.
- Xie J, Xu K, Cai Z, Chen M, Jiang Y, Ye J, et al. Efficacy and safety of first-line PD-L1/PD-1 inhibitors in limited-stage small cell lung cancer: a multicenter propensity score matched retrospective study. Transl Lung Cancer Res [Internet]. AME Publishing Company; 2024 [cited 2025 Sept 12];13. https://doi.org/10.21037/tlcr-24-24.
  - ○ This study on 150 patients has a long minimum follow-up time of 21 months and reports no significant difference in the rate of grade ≥ 3 TRAEs between ICI plus Chemotherapy and Chemotherapy alone, as well as Anti-PD-1 and Anti-PD-L1 inhibitors when combined with chemotherapy.
- Yamanaka Y, Okuno Y, Kamisako K, Okazaki Y, Nakanishi K, Sanada Y, et al. Efficacy and Safety Evaluation of Immune Checkpoint Inhibitors in Combination With Chemotherapy for Extensive Small Cell Lung Cancer: Real-World Evidence. Cancer Med. 2024;13:e70480. https://doi.org/10.1002/cam4.70480.
  - ○ This real-world study (*n* = 100) found no significant differences in grade ≥ 3 TRAEs and irAEs between atezolizumab and durvalumab when combined with chemotherapy.
- Zhang J, Wu Z, Wang S, Sun Y, Wu J, Wang D, et al. Efficacy of PD-(L)1 inhibitors plus anlotinib in the second-line treatment of extensive-stage small cell lung cancer. BMC Cancer. 2025;25:1070. https://doi.org/10.1186/s12885-025-14458-5.
  - ○ This large real-world study (*n* = 389), involving one of the few analyses with anlotinib in a comparator group, stands out for its larger sample size.

## Supplementary Information

Supplementary Material 1.

## Data Availability

All materials used in this systematic review have been fully referenced and are available within the paper and its Supplementary Information.
