# Supplementary material for: Real-World Safety of Immune Checkpoint Inhibitors in Small Cell Lung Cancer: A Systematic Review of Comparative Cohort Studies
Source: Curr Oncol Rep. 2026 Mar 26;28(1):27. doi: 10.1007/s11912-026-01774-7 (PMC13021803; doi:10.1007/s11912-026-01774-7)
Supplement: Supplementary file 1 — Supplementary Material 1. [file 11912_2026_1774_MOESM1_ESM.pdf]

Real-World Safety of Immune Checkpoint Inhibitors in Small Cell Lung Cancer: A Systematic Review of Comparative Cohort Studies

Juhee Koo, Chin Hang Yiu, Hieu T. Le, Kevin Winardi, Edwin C.K. Tan, Christine Y. Lu

Current Oncology Reports

Corresponding author: Christine Y. Lu

Email: christine.lu@sydney.edu.au

Affiliations:

The University of Sydney School of Pharmacy, Camperdown, Sydney, New South Wales, Australia

Kolling Institute, Faculty of Medicine and Health, The University of Sydney and the Northern Sydney Local Health District, St Leonards, Sydney, New South Wales, Australia

Department of Pharmacy, Royal North Shore Hospital, St Leonards, Sydney, New South Wales, Australia

Supplementary Table S1: Changes to PROSPERO protocol with reasons

Number	Protocol	Change(s)	Reason(s)
**1**	Patients with any type of lung cancer were included.	Only patients with small cell lung cancer were included.	The clinical course, biological behaviour, and treatment paradigms for SCLC differ significantly from those of NSCLC. Given these distinctions, it is not methodologically appropriate to generalise safety outcomes or treatment-related adverse events across both subtypes. As such, the scope of this review was refined to focus exclusively on SCLC to ensure clinical relevance, improve the specificity of the findings, and enhance the applicability of the results to SCLC patient populations.
**2**	The search was conducted from database inception to 20th March 2025.	The search was conducted from database inception to 21st July 2025.	To ensure that the review captures the most current evidence, the literature search was extended to include studies published up to 21st July 2025. This change was made to incorporate any newly published articles, thereby enhancing the completeness and relevance of the review findings.
**3**	The search strategy was not lung cancer-specific and included ICI names and a theme for 'risk factors'.	The search strategy included a theme for lung cancer and did not include specific ICI names or 'risk factors'.	The inclusion and exclusion criteria were modified to ensure the search was more specific to the target population—patients with SCLC—while avoiding overly restrictive parameters that might exclude potentially relevant real-world studies. This adjustment aimed to balance specificity and sensitivity in the search strategy, thereby improving the relevance and comprehensiveness of the review.
**4**	The relevant safety outcomes included incidence rates, hazard ratios, and/or odds ratios.	The relevant safety outcomes included incidence rates only.	Hazard ratios and odd ratios were not reported in the included studies and, as a result, could not be extracted or analysed in this review.

Supplementary Table S2: Full Search Strategy

**MEDLINE**	
1	Lung Neoplasms/
2	Lung cancer*.mp.
3	1 or 2
4	Immune Checkpoint Inhibitors/
5	(immune checkpoint inhibitor* or checkpoint inhibit* or immunotherap*).mp.
6	4 or 5
7	(immune-related adverse event* or irAE*).mp.
8	(side effect* or adverse drug event* or adverse drug react* or toxic* or safety).mp.
9	7 or 8
10	Electronic Health Records/ or Medical Records/
11	Databases, Pharmaceutical/
12	(medic* record* or electronic health record*).mp.
13	(claim* or claim* data* or drug* claim* or medic* claim* or administrative claim* or administrative data*).mp.
14	(health* system* or health* data* or dispens* record* or medic* list* or medical record* or pharmacy data* or prescription data* or hospital record* or hospital data* or real world).mp.
15	Comparative Study/
16	10 or 11 or 12 or 13 or 14 or 15
17	3 and 6 and 9 and 16
**EMBASE**	
1	lung cancer/
2	lung cancer*.mp.
3	1 or 2
4	immune checkpoint inhibitor/
5	(immune checkpoint inhibitor* or checkpoint inhibit* or immunotherap*).mp.
6	4 or 5
7	(immune-related adverse event* or irAE*).mp.

8	(side effect* or adverse drug event* or adverse drug react* or toxic* or safety).mp.
9	7 or 8
10	electronic medical record/ or electronic health record/ or medical record/
11	drug database/
12	(claim* or claim* data* or drug* claim* or medic* claim* or administrative claim* or administrative data*).mp.
13	(health* system* or health* data* or dispens* record* or medic* list* or medical record* or electronic health record* or pharmacy data* or prescription data* or hospital record* or hospital data* or real world).mp.
14	comparative study/
15	10 or 11 or 12 or 13 or 14
16	3 and 6 and 9 and 15
**SCOPUS**	
TITLE-ABS-KEY(("lung cancer*" OR "lung neoplasm*") AND ("immune checkpoint inhibitor*" OR "checkpoint inhibit*" OR immunotherap*) AND ("immune related adverse event*" OR irae* OR "side effect*" OR "adverse drug event*" OR "adverse drug react*" OR toxic* OR safety) AND ("claim*" OR "administrative claim*" OR "administrative data*" OR "claim* data*" OR "pharmacy data*" OR "prescription data*" OR "health* data*" OR "health system*" OR "medical record*" OR "medical chart*" OR "electronic health record*" OR "hospital record*" OR "hospital data*" OR "real world" OR "comparative study"))	
**CINAHL**	
("lung cancer*" OR "lung neoplasm*" OR "lung tumor*") AND ("immune checkpoint inhibitor*" OR "checkpoint inhibit*" OR immunotherap*) AND ("immune-related adverse event*" OR irae* OR "side effect*" OR "adverse drug event*" OR toxic* OR safety) AND (("pharmacy data*" OR "prescription data*" OR "health* data*" OR "health system*" OR "medical record*" OR "medical chart*" OR "electronic health record*" OR "hospital record*" OR "hospital data*") OR (claim* OR "administrative claim*" OR "administrative data*" OR "claim* data*") OR ("real world" OR "comparative study"))	

Supplementary Table S3: Example calculation for incidence rate per capita

<table>
<tr><th colspan="2">Zhao et al. (2025)</th></tr>
<tr><td>ICI plus chemotherapy group (n=82)

<u>Grade ≥3 TRAEs</u>
• Leukopenia (n=16)
• Neutropenia (n=30)
• Anaemia (n=6)
• Thrombocytopenia (n=9)
• Lymphopenia (n=18)
• Transaminase increase (n=0)
• Creatinine increase (n=0)
• Nausea or vomiting (n=0)
• Diarrhoea (n=0)
• Constipation (n=0)

1. 16 + 30 + 6 + 9 + 18 + 0 + 0 + 0 + 0 + 0 = 79 total grade ≥3 TRAEs
2. 79/82 = 0.96 grade ≥3 events per capita</td><td>Chemotherapy alone group (n=53)

<u>Grade ≥3 TRAEs</u>
• Leukopenia (n=6)
• Neutropenia (n=17)
• Anaemia (n=2)
• Thrombocytopenia (n=4)
• Lymphopenia (n=12)
• Transaminase increase (n=0)
• Creatinine increase (n=0)
• Nausea or vomiting (n=0)
• Diarrhoea (n=0)
• Constipation (n=0)

1. 6 + 17 + 2 + 4 + 12 + 0 + 0 + 0 + 0 + 0 = 41 total grade ≥3 TRAEs
2. 41/53 = 0.77 grade ≥3 events per capita</td></tr>
<tr><td colspan="2">Similar calculations were conducted for the following studies: Qin et al. (2024), Wan et al., (2023), Xu et al. (2024), Yamanaka et al. (2024), J, Zhang et al. (2025), and Q, Zhang et al. (2025)</td></tr>
</table>

Supplementary Table S4: Newcastle-Ottawa Quality Assessment for cohort studies (n=20)

Author (Year)	Selection				Comparability	Outcome			Quality (AHRQ standards)
	Representativeness of exposed cohort (Max: 1★)	Selection of the non-exposed cohort (Max: 1★)	Ascertainment of exposure (Max: 1★)	Demonstration that outcome of interest was not present at start of study (Max: 1★)	Comparability of cohorts on the basis of design and analysis (Max: 2★)	Assessment of outcome (Max: 1★)	Was follow-up long enough for outcomes to occur† (Max: 1★)	Adequacy of follow-up cohorts‡ (Max: 1★)	
He et al. (2024)	★	★	★	★	– No confounders were adjusted. Only included a statement that differences between cohorts were well balanced.	★	★	★	Poor
Lamy et al. (2024)	★	★	★	★	– No confounders were adjusted for safety data, only for efficacy data.	★	★	★	Poor
Peng et al. (2025)	★	★	★	– Outcome of interest may have been ongoing before disease progression.	– No confounders were adjusted. Only included a statement that differences between cohorts were well balanced.	★	– Shortest follow-up time may have been 8 months.	★	Poor
Qin et al. (2024)	★	★	★	★	– No confounders were adjusted. Only included a statement that differences between cohorts were not statistically significant.	★	– Shortest follow-up time was 3 months.	★	Poor
Qiu et al. (2023)	★	★	★	★	– No confounders were adjusted. Only cohort baseline characteristics were reported in Table 1.	– No description of safety assessment.	– Shortest follow-up time may have been 10 months.	★	Poor
Qu et al. (2022)	★	★	★	★	– No confounders were adjusted. Only cohort	★	– Shortest follow-up was 2 months.	★	Poor

					baseline characteristics were reported in Table 1.				
Vince et al. (2024)	★	★	★	★	– No confounders were adjusted. Only included a statement that differences between cohorts were not statistically significant.	– No description of safety assessment.	– No information on follow-up.	★	Poor
Wang et al. (2024)	★	★	★	★	– No confounders were adjusted. Only included a statement that baseline characteristics were comparable.	★	– Shortest follow-up time may have been 7 months.	★	Poor
Wang et al. (2023)	★	★	★	★	– No confounders were adjusted. Only included a statement that baseline characteristics were well balanced.	★	– Shortest follow-up time may have been 8 months.	★	Poor
Wan et al. (2023)	★	★	★	★	– No confounders were adjusted. Only included a statement that baseline characteristics were similar.	★	– No information on follow-up.	★	Poor
Xie et al. (2024)	★	★	★	★	– Propensity score matching was not conducted for data on trAEs.	– No description of safety assessment.	★	★	Poor
Xu et al. (2024)	★	★	★	★	– No confounders were adjusted. Only cohort baseline characteristics were reported in Table 1.	★	– Shortest follow-up time may have been 2 months.	★	Poor
Yamanaka et al. (2024)	★	★	★	★	– No confounders were adjusted. Only cohort baseline characteristics were reported in Table 1.	★	– The shortest follow-up time was 0.7 months.	★	Poor

J Zhang et al. (2025)	★	★	★	★	– Propensity score matching was used, but confounders adjusted for are not stated.	★	★	★	Poor
Q Zhang et al. (2025)	★	★	★	★	– No confounders were adjusted. Only included a statement that differences between cohorts were not statistically significant.	★	– No information on follow-up.	★	Poor
Zhang et al. (2024)	★	★	★	★	– No confounders were adjusted. Only included a statement that differences between cohorts were not statistically significant.	★	– No information on follow-up.	★	Poor
Zhao et al. (2025)	★	★	★	★	– No confounders were adjusted. Only included a statement that the distributions of all variables except treatment modality did not differ significantly.	★	★	★	Poor
Zhou et al. (2025)	★	★	★	★	★★ Propensity score matching, with covariates of age, ECOG performance status, smoking history, number of metastatic sites, and history of chest and brain radiotherapy	★	– Shortest follow-up time may have been 9 months.	★	Good
Zhu et al. (2025)	★	★	★	★	– No confounders were adjusted. Only included a statement that differences between cohorts were not statistically significant.	★	★	★	Poor
Zou et al. (2023)	★	★	★	★	– No confounders were adjusted. Only included a statement that baseline	★	– Information on follow-up	★	Poor

					characteristics were balanced.		duration not included.		
